# Mechanical and Electrical Properties of Cementitious Composites Reinforced with Multi-Scale Carbon Fibers

**DOI:** 10.3390/ma18081830

**Published:** 2025-04-16

**Authors:** Nueraili Maimaitituersun, Jing Wang, Danna Wang, Zuojun Ning

**Affiliations:** 1 School of Transportation, Kashi University, Kashi 844000, China; nurali2023@ksu.edu.cn; 2 Tianjin Urban Road Facility Inspection Center, Tianjin 300190, China; stduwangjing@gmail.com; 3 College of Civil Engineering and Architecture, Zhejiang University of Water Resources and Electric Power, Hangzhou 310018, China

**Keywords:** cementitious composites, short-cut carbon fibers, carbon nanotubes, uniaxial tensile properties, electrical conductivity

## Abstract

Carbon fibers, with high modulus of elasticity, tensile strength, and electrical conductivity, can modify the mechanical and electrical properties of cementitious composites, facilitating their practical application in smart infrastructure. This study investigates the effects of carbon nanofibers (including carbon nanotubes, a special type of carbon nanofibers) and micron carbon fibers with different aspect ratios and surface treatments on the uniaxial tensile and electrical properties of cementitious composites. The results demonstrate that appropriate carbon fiber doping markedly improves the uniaxial tensile strength of cementitious composites, with enhancement effects following a gradient trend based on a geometric scale: carbon nanotubes (CNTs) < carbon nanofibers (CNFs) < short-cut carbon fibers (CFs). Hydroxyl-functionalized multi-walled carbon nanotubes (MWCNTs) form continuous conductive networks due to surface active groups (-OH content: 5.58 wt.%), increasing the composite’s electrical conductivity by two orders of magnitude (from 3.56 × 10^8^ to 2.74 × 10^6^ Ω·cm), with conductivity enhancement becoming more pronounced at higher doping levels. Short-cut CFs also improve conductivity, with longer fibers (6 mm) exhibiting a 12.4% greater reduction in resistivity. However, exceeding the percolation threshold (0.5–1.0 vol.%) leads to limited conductivity improvement (<5%) and mechanical degradation (8.7% tensile strength reduction) due to fiber agglomeration-induced interfacial defects. This study is a vital reference for material design and lays the groundwork for self-sensing cementitious composites.

## 1. Introduction

As a critical material in modern construction, cementitious composites face challenges such as environmental erosion, fatigue, and material aging. This constantly elevates its properties’ requirements, including high strength, toughness, durability, and volumetric stability. In recent years, studies have been conducted to improve the toughness, crack resistance, and overall durability of cementitious composites by introducing different fiber materials, for example, steel fibers, polypropylene fibers, glass fibers, or carbon fibers [1,2,3,4,5,6]. The introduction of fibers not only improves the fracture mode of cementitious composites from brittle to ductile, but also increases the cracking strength, peak tensile strength, and energy absorption capacity of composites by bridging cracks [7,8,9,10,11,12]. Carbon fiber, as a high-performance fiber, has excellent properties such as high tensile strength, high modulus of elasticity, high creep resistance, low specific gravity, being non-corrosive, low electrical resistivity, and high thermal conductivity; therefore, it is commonly used to strengthen and toughen cementitious composites [6,7,13]. In addition, carbon fiber can also be incorporated into composites as a conductive phase to enhance its electrical conductivity [14]. As a result, a lot of research in recent years has been carried out around the mechanical and electrical properties of carbon fiber-reinforced cementitious composites (CRCCs).

Studies have shown that the scale and surface properties of fibers have significant effects on the bond strength of cementitious composites [3]. CNTs, as nano-scale fibrous materials characterized by hollow structures, high aspect ratios, and exceptional mechanical properties, effectively suppress the initiation and propagation of nano-scale cracks in cementitious composites. At a dosage of 0.02% to 0.5% by weight, CNTs enhance compressive strength by 10–40% and flexural strength by 9.3–43.38%, demonstrating their significant reinforcing potential in such matrices [15]. Further research reveals that within the dosage range of 0.25–0.50 wt.%, adjusting the aspect ratio (L/D = 1000–1500) of CNTs significantly enhances the tensile strength (up to 31.94%), energy absorption capacity (46.39%), and deformation performance of RPC, while optimizing the pore structure and interfacial bonding. Notably, larger L/D ratios improve toughness through bridging effects and crack suppression, whereas smaller L/D ratios prioritize enhancement of Young’s modulus [16]. Additionally, CNTs’ unique nano-scale hollow structure and high aspect ratio confer outstanding electrical conductivity. Their tubular architecture facilitates efficient axial electron migration, forming continuous conductive networks that drastically reduce composite resistivity [17,18,19,20,21,22]. For instance, Luo et al. [15] demonstrated that incorporating 2 wt.% CNTs decreased the resistivity of cementitious composites to 1.83 kΩ·cm^−1^, a 99.8% reduction compared to ordinary concrete while enabling self-sensing functionality. However, CNTs’ high specific surface area and strong inter-tube van der Waals forces lead to poor dispersion in cement matrices, and weak interfacial bonding limits their reinforcement efficiency [23,24]. To address this, multi-walled carbon nanotubes (MWCNTs) are preferred over single-walled CNTs (SWCNTs) due to their lower production cost, reduced agglomeration tendency, and enhanced dispersion stability, which collectively improve composite performance [18,25]. Surface modification techniques, such as chemical functionalization, are widely employed to enhance CNT dispersion [19,26]. Functionalized CNTs, through the introduction of active surface groups (e.g., carboxyl or hydroxyl groups), exhibit improved compatibility with the matrix, resulting in more stable dispersion states, as validated by multiple studies [27,28,29].

In addition, CNFs are sub-micron-scale carbon fiber materials, which not only have the characteristics of small size and large specific surface area of nanomaterials but also have high stability, high electrical conductivity, and high elastic modulus [30]. Studies indicate that even at low dosages (e.g., 0.05–0.15 wt.%), CNFs can significantly enhance both the mechanical properties and functionality of cement-based composites. Guan Fenglin [15] found that incorporating 0.05 wt.% CNFs maximized the 28-day flexural strength improvement in cement mortar, while a 0.1 wt.% dosage notably increased the 28-day compressive strength. This strengthening effect stems from CNFs’ unique mechanisms: their high aspect ratio and rigid structure effectively bridge microcracks and inhibit crack propagation. Simultaneously, CNFs refine the densification of the microstructure by filling pores and optimizing the interfacial bonding between the matrix and fibers. Additionally, the electrical conductivity of CNFs highlights their potential in smart construction materials. Low CNF dosages (e.g., 0.1 wt.%) not only establish continuous conductive networks to reduce composite resistivity [31,32,33,34] but also impart self-sensing capabilities, enabling real-time monitoring of stress, strain, and damage evolution. For example, acid-treated CNFs can enhance piezoresistive sensitivity by 150%, while maintaining stable performance under freeze–thaw cycles and chloride ion ingress [35]. Compared to carbon nanotubes (CNTs), CNFs’ micron-scale dimensions facilitate more uniform dispersion in cement matrices, avoiding agglomeration issues caused by CNTs’ high specific surface area and van der Waals forces [23,24]. Moreover, CFs, a microcrystalline graphite material with a large aspect ratio, low density, high modulus of elasticity, and excellent electrical conductivity [36], have attracted a lot of attention in reinforcing cementitious composites due to their excellent mechanical and conductivity properties [37,38,39].

Due to the high elastic modulus, high axial tensile strength, and high electrical conductivity, carbon fibers enhance the mechanical and functional properties of cementitious composites, such as flexural, tensile, and compressive strengths, stiffness, electrical conductivity, and sensory sensitivity, without changing the chemical properties of the composites. Current research has focused on the mechanical properties of CRCCs, especially compressive, flexural and splitting strengths, while the axial tensile properties are less studied [37,40]. The tensile properties of CRCCs are affected by a combination of factors, among which the doping and size of carbon fiber are particularly critical [41,42]. Therefore, an in-depth study on the influence mechanism of carbon fiber parameters on the tensile properties of cementitious composites is of great significance in improving the safety and reliability of concrete structures. Meanwhile, smart cementitious composites with self-adaptive, self-diagnostic and self-healing capabilities can predict and prevent the internal damage of composites, thus reducing engineering accidents. In addition, the electrical conductivity of smart cementitious composites is the key index to realize these functions. Consequently, studying and improving cementitious composites’ electrical properties is essential to promote innovative development.

**Figure 1:**
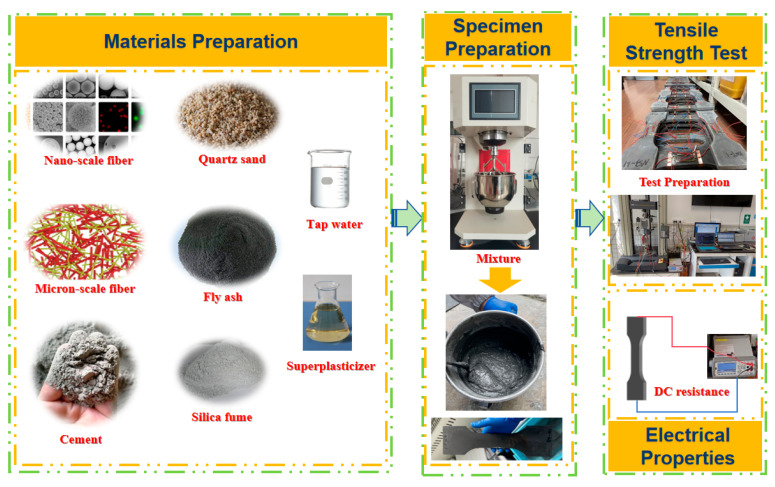
The main research content of this paper.

In this study, CRCCs were prepared by combining functionalized and unfunctionalized MWCNTs, CNFs, and CFs at various scales to study the contributions of nano-scale and macro-scale carbon fibers to the axial tensile strength and electrical properties of the composites. The main research content of this paper is presented in Figure 1. Among them, the functionalized MWCNTs primarily include carboxylated MWCNTs and hydroxylated MWCNTs. The effects of the scale and doping of carbon fibers on the uniaxial tensile strength and fracture strain energy were investigated through uniaxial tensile tests. Meanwhile, the impact of nano-scale fibrous materials and CFs on the electrical conductivity of the composites was analyzed, with a focus on exploring their enhancement effects and the underlying conductivity mechanisms.

## 2. Experimental Design

### 2.1. Raw Materials

In this study, the primary raw materials for the preparation of CRCC specimens were P·Ι 42.5 Portland cement (C), silica fume (SF), class I fly ash (FA), quartz sand (QS), a high-efficiency water reducing agent, tap water (W), naphthalene sulfonate superplasticizer (NSF), nano-scale fibrous materials (including CNTs, a special type of CNFs), and CFs. The composition of C is separately shown in Table 1 and Table 2 [43]. SF exhibits an average particle size of approximately 150 nm, a bulk density of 150–700 kg/m^3^, a specific gravity of 2.2–2.3 g/cm^3^, and a high specific surface area of 15–30 m^2^/g. Its nano-scale particle characteristics and amorphous silica structure confer significant advantages in nano-scale reinforcement and high-performance composite materials [43]. The waste FA powder from coal-fired power plants was used as a cementitious material to reduce the amount of cement, while simultaneously improving the fluidity of the freshly mixed slurry. The loss on ignition, fineness, and water demand ratio of FA were 2.74%, 9.9% (residue on a 45 μm square hole sieve), and 93%, respectively, and its chemical compositions are listed in Table 3. The particle size of QS ranges from 0.12 mm to 0.83 mm, with a SiO_2_ content of 99%~99.5% and a Fe_2_O_3_ content of ≤0.005%. The 3301E type polycarboxylate superplasticizer was used, with a water reduction rate and a solid content of about 30% and 45%, respectively. It should be noted that the characterization information of the raw materials all comes from suppliers. The carbon fiber materials selected were elongated hydroxyl-functionalized MWCNTs (MHI), elongated carboxyl-functionalized MWCNTs (MCI), thick and long MWCNTs (M5), industrial MWCNTs (IM), PR-19-XT-HHT-type CNFs (CNF19), PR-24-XT-HHT type CNFs (CNF24), and CFs with lengths of 3 mm (CF3) and 6 mm (CF6), respectively. Their main properties are shown in Table 4. The selection criteria of these fibers were established based on their advantages, in which MHI employs hydroxyl functional groups to optimize alkaline dispersion stability [44], MCI utilizes carboxyl groups to enhance interfacial chemical bonding [16], while CNF24/CNF19 prioritizes high modulus characteristics for load transfer optimization and adopts a whisker-like structure to improve electrical conductivity [45,46,47]. CF3/CF6 were selected to investigate macroscopic mechanical response variations through different fiber lengths; IM serves as an unmodified control group to quantify functionalization effects, and M5 is included to analyze the effects of aspect ratio on performance. By integrating the multi-scale synergistic effects of nano-scale fibers (interfacial reinforcement and conductive network construction) and macro-scale fibers (crack-bridging mechanisms), this study systematically elucidates the regulatory mechanisms of carbon fibers on the tensile strength, fracture toughness, and electrical properties of the composites.

### 2.2. Ratio and Specimen Preparation

#### 2.2.1. Mixing Ratios

In this study, the mix ratios for cementitious composites recommended in the literature [43] were adopted, and the mass ratio of binder materials (C + FA) to SF and QS was 1:0.25:1.1. Specifically, the binder materials consisted of 20% FA replacing cement by mass (i.e., for a total binder mass of 1 unit, C accounted for 0.8 units and FA for 0.2 units). The QS was composed of coarse (0.5–1.0 mm), medium (0.25–0.5 mm), and fine (0.12–0.25 mm) particles mixed in equal proportions by mass to achieve gradation optimization. All material ratios were calculated based on the total mass of the binder materials (1 unit): SF was added at 0.25 units, NSF was added at 0.011 units, and the water-to-cement ratio was set to 0.375. Combined with the particle size of raw materials and the optimum dosage of different nano-scale fibrous materials in relevant literature [16], the mixing ratios of CRCCs filled with MHI/MCI/M5/IM/CNF19/CNF24, which are used in this study, are shown in Table 5. PC in Table 5 denotes cementitious composites without carbon fiber doping. In addition, the volumetric doping of the two CFs (CF3 and CF6) was 0.50 vol.%, 1.0 vol.%. and 1.5 vol.%, respectively. In addition, three specimens were prepared in duplicate for each set of CRCC specimens. The specimens were labeled using a “fiber type-dosage” nomenclature (e.g., CF3-0.5 denotes 3 mm chopped carbon fibers with a 0.5 vol% dosage). Three parallel specimens were prepared for each group, and test results are reported as mean values.

#### 2.2.2. Specimen Preparation

**Figure 2:**
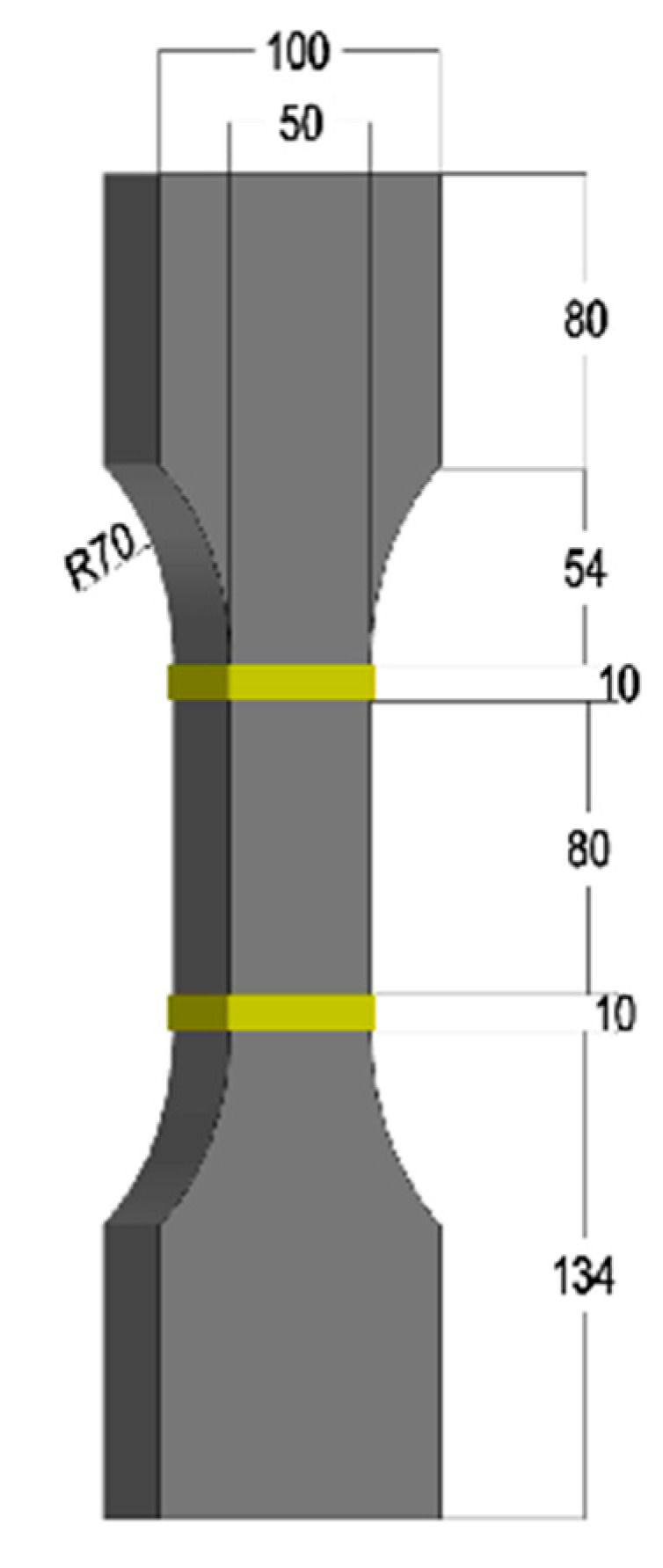
Specimen dimension (Unit: mm).

In this study, the uniaxial tensile test was carried out using a “dog bone” shaped specimen with a thickness of 15 mm, and the dimensions of the specimen are shown in Figure 2. To ensure the uniform distribution of nanofillers and fiber fillers, different fabricating methods were used for the CRCC specimens with nano-scale fibrous materials and CRCC specimens with CFs.

(1) Specimen preparation process of CRCC with nano-scale fibrous materials

Firstly, water, a high-efficiency water reducing agent, and nanofillers were added together. After appropriate agitation, the mixture was ultrasonicated with a probe ultrasonic instrument for 5 min to ensure a uniform distribution of nanofillers in the slurry. During this process, the power of the ultrasonic instrument was set to 400 W. Then, SF was poured into the well-dispersed mixture and stirred at a low speed for 60 s. Thirdly, C and FA were added and sequentially mixed at a low speed for 60 s and a high speed for 120 s. Subsequently, the QS was poured and then mixed in turn at a low speed for 60 s and a high speed for 120 s. Next, the homogeneous slurry after mixing was poured into the oiling mold and placed on a vibration table for 60 s to obtain a dense slurry. Afterward, the surface of the slurry was smoothed. The specimens were then placed in a standard curing chamber at a temperature of 20 °C and a relative humidity of 95% for 24 h for demolding. Finally, they were further cured in room temperature water for 28 days and then dried in air for 24 days.

(2) Specimen preparation process of CRCC with CFs

The only difference in the specimen preparation process between CRCCs with CFs and CRCCs with nano-scale fibrous materials was that those with CFs did not require ultrasonic treatment and could be directly mixed with SF and stirred.

### 2.3. Performance Test Methods

#### 2.3.1. Uniaxial Tensile Test

**Figure 3:**
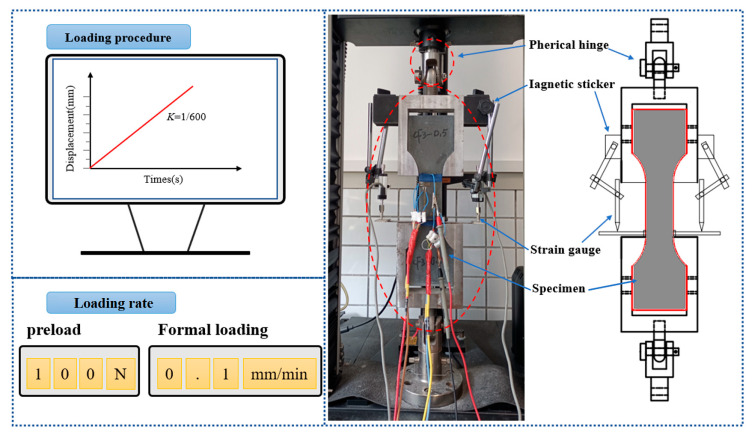
Schematic diagram of the uniaxial tensile test.

The uniaxial tensile test in this study was carried out in accordance with the GB/T 50081-2019 standard [48] and the CECS 13-2009 standard [49]. The loading test was performed using an electronic universal testing machine (UTM, Model Instron5567, Instron Corporation, Boston, MA, USA) with a range of 30 kN. The test system employs a bidirectional spherical hinge linkage mechanism to achieve neutral load transfer, with specimens secured by bidirectional dedicated fixtures. During installation, transverse adjustment bolt sets are symmetrically tightened to ensure precise alignment between the loading axis and the specimen’s geometric centerline. In the preloading phase, a closed-loop controlled preload force of 100 N is applied at a deformation rate of 0.05 mm/min, while bilateral extensometer data are synchronously analyzed to verify alignment neutrality. The system automatically halts and triggers a reset calibration procedure when strain deviation exceeds 2%, effectively eliminating system clearance and compensating for elastic deformation. The formal loading phase maintains a constant rate of 0.1 mm/min, with experimental device configurations and loading procedures illustrated in Figure 3. As shown in Figure 3, two longitudinal and two transverse strain gauges were arranged in the middle effective zone of the specimen, and two clamped extensometers were placed at the left and right ends to measure the tensile deformation of the specimens. The strain and deformation of the specimens were collected by the dynamic digital acquisition instrument (DSA, Model DH3820N, Donghua Testing Technology, Taizhou, China). The acquisition frequency of the universal testing machine and the data acquisition instrument were set to 10 Hz to ensure the synchronization of strain, deformation, and load change data.

The tensile strength of a CRCC is expressed as the maximum tensile force that can be sustained by the cross-sectional area of the specimen and can be calculated using Equation (1). Equation (2) can then be used to determine the tensile strength increase rate for each group of specimens, expressed as a percentage. This ratio effectively highlights the changes in the tensile strength of the composite specimens, as influenced by different volumes of carbon fiber incorporation. The ratio is expressed as follows:

(1) $$f_{t}=F_{t}/bh$$

(2) $$R_{t}=(f_{t}-f_{o})/f_{o}\times 100\%$$

where $f_{t}$ and $\;f_{o}$ are the tensile strength of cementitious composites with and without different carbon fiber dosage, respectively. $F_{t}$ represents the ultimate load. *b* and *h* separately denote the width and thickness of the specimen, which are 50 mm and 15 mm in this experiment. $R_{t}$ is the tensile strength increase rate (%) of the CRCC specimen.

**Figure 4:**
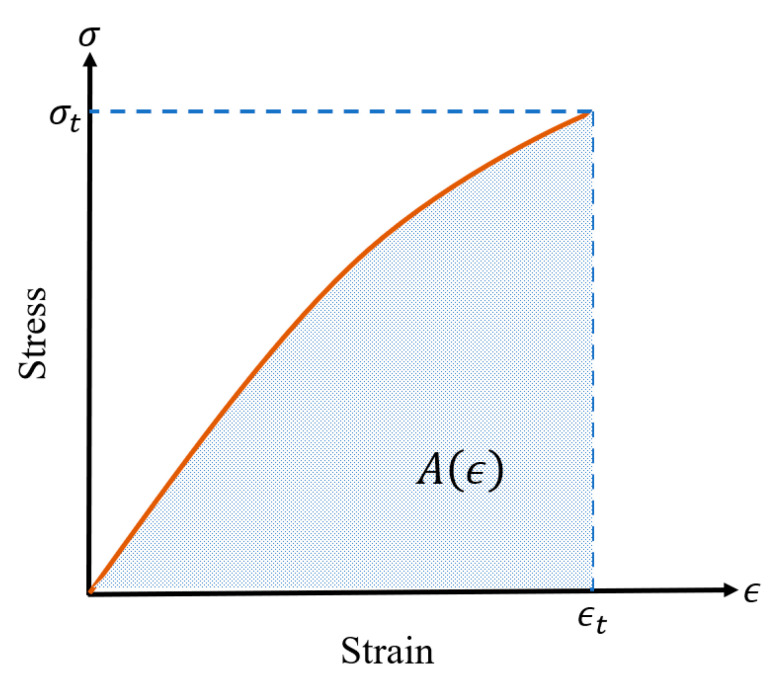
Stress–Strain variation curve.

Strain energy is the potential energy stored within a structure, manifested through stress and strain, and can be represented by the area under the stress–strain curve of the material. Two commonly used test methods for testing strain energy are the direct tensile test and the three-point bending test. Since the bending test of a notched specimen is more spartan, many researchers use the three-point bending test with a notched beam to determine the strain energy of cementitious composites [50,51,52]. In contrast to the three-point bending test, the direct tensile test is ideal for testing the fracture properties of cementitious composites as it not only capable of plotting the stress–strain curve (as shown in Figure 4) but also provides additional mechanical property data such as the tensile strength. The value of fracture strain energy can be obtained from the following Equation (3):

(3) $$A\left(\epsilon \right)=\int_{0}^{\epsilon_{t}}\sigma_{x}d\epsilon_{x}$$

where $A\left(\epsilon \right)$ is the strain energy, calculated by the area under the stress–strain curve in the direct tensile test, according to Figure 4. $\epsilon_{t}$ denotes the ultimate strain of the specimen. $\epsilon_{x}$ is strain corresponding to a moment in the axial tensile test, and $\sigma_{x}$ is the corresponding stress.

#### 2.3.2. Conductivity Test

**Figure 5:**
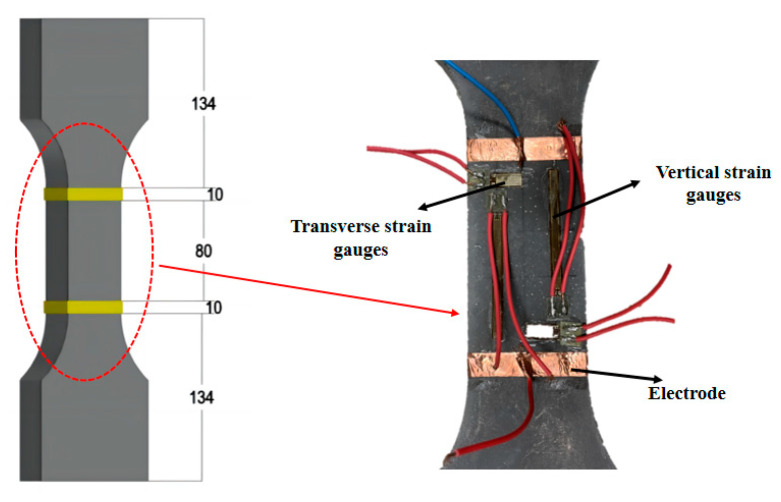
Electrode material and layout diagram (Unit: mm).

To study the variation rule of electrical resistance of CRCCs with filler doping, this paper adopts the two-electrode method to measure the direct current (DC) resistance of composite specimens after 28 days of curing. The electrode system was fabricated using brass conductive adhesive tape (with the width of 10 mm and the thickness of 0.2 mm) as the interfacial medium, exhibiting a contact resistance of 5.0 mΩ and an adhesion strength of 3.8 N/10 mm. A dual-electrode DC resistance measurement system was established in compliance with the GB/T 3048.2-2007 standard [53], incorporating a Keithley 2100 precision multimeter as the core acquisition unit. The electrode configuration and material arrangement are detailed in Figure 5. It is noteworthy that the surface moisture of the specimen should be wiped dry first, and then the specimen should be dried in the air for 12 h before measuring the resistance. The resistivity can be calculated by substituting the measured values into Equation (4), which is as follows:

(4) ρ=RS/L

where  ρ is the volume resistivity of the specimen, and *R* is the resistance of the specimen. *S* and *L* represent the cross-sectional area and the length of the resistance test portion of the specimen, respectively.

## 3. Results and Discussion

### 3.1. Modes of Failure

**Figure 6:**
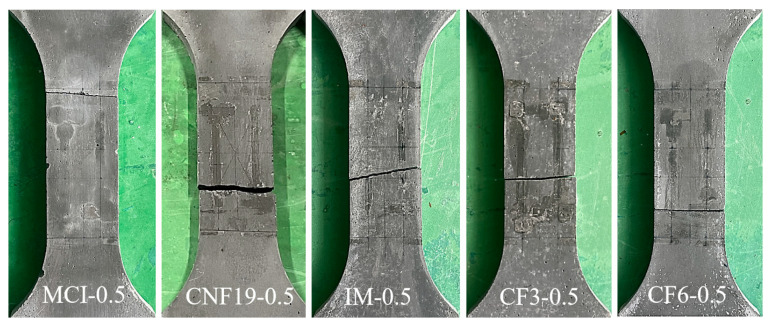
Typical failure modes of CRCCs in uniaxial tensile test.

The test results indicate that within the effective region of the specimen, as the load increases, the crack gradually propagates towards both ends of the specimen, ultimately forming a continuous I-shaped crack. The final uniaxial tensile damage patterns of CRCC specimens with different carbon fibers are provided in Figure 6. Consistent with findings from reference [16], this study observes that both control specimens and carbon nanofiber-reinforced CRCC specimens exhibit typical brittle fracture characteristics, featuring well-defined crack profiles with minimal spalling. This aligns with conclusions from references [16,54] regarding nanofiber-reinforced materials, confirming that while nano-scale reinforcements provide micro-level crack bridging, their effectiveness in improving macroscopic fracture behavior remains limited. Notably, specimens reinforced with CF display distinctly different failure modes. Although they similarly develop main through-thickness cracks with clear contours, they show significantly reduced spalling, consistent with the results in reference [55]. The difference stems from the more efficient three-dimensional bridging system formed by micro-scale short-cut fibers, where the fiber pull-out mechanism dissipates additional fracture energy, thereby substantially mitigating the brittle nature of the composites [55]. This study confirms that the toughening effect of micro-scale short-cut fibers markedly surpasses that of nano-scale fibers, which aligns with the current research consensus on fiber-reinforced cementitious composites.

### 3.2. Stress–Strain Curves

**Figure 7:**
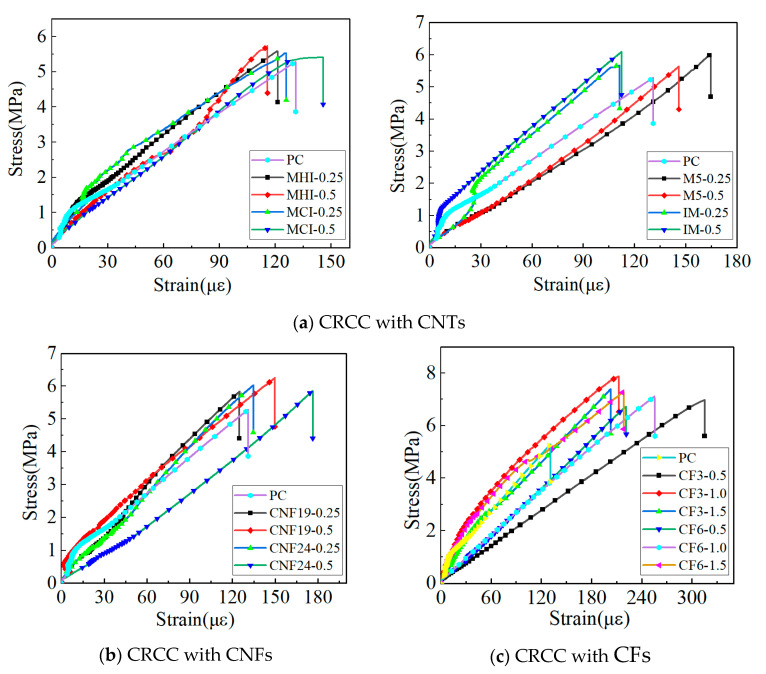
Load–deformation curves of CRCCs under uniaxial tensile loads.

Figure 7 illustrates the stress–strain response characteristics of carbon nanofiber-reinforced cementitious composites (CRCCs) under uniaxial tensile loading, with the peak stress and ultimate strain values for each test group detailed in Table 6. The results (Figure 7a) indicate that specimens with high/low dosages of the same type of carbon nanofibers exhibit similar mechanical response patterns, and the stress–strain curve morphologies of CRCCs with different nanofibers show no significant deviation compared to the control group. Specifically, the stress–strain curves of MHI- and MCI-doped specimens completely overlap with those of the control group, confirming their inability to alter the brittle fracture characteristics of the composites, consistent with findings for non-functionalized carbon nanotubes reported in the literature [16]. Notably, the stress–strain curves of the M5-doped specimens lie entirely below the control group, with a total deformation increase of approximately 15%, while the IM-doped specimens display an anomalous stiffening behavior (curves above the control group, total deformation reduced by 22%). This contradictory behavior can be attributed to the coupled effects of carbon nanotube geometric properties and dispersion states. Similar results were reported in reference [18], showing that thick-walled carbon nanotubes with high aspect ratios (e.g., M5 with the outer diameter of 20–30 nm and the length of 10–30 μm) can significantly enhance toughness through three-dimensional bridging under ideal dispersion conditions. However, in practical preparation, van der Waals forces induce agglomeration, increasing matrix porosity by about 8.7% and thereby diminishing reinforcement efficiency. In contrast, industrial-grade carbon nanotubes (IM with the length of 10–20 μm), despite their larger aspect ratios, exhibit severely limited interfacial bonding performance due to the absence of surface functionalization and residual impurities. These findings reveal the synergistic regulatory mechanism of nanofiber intrinsic properties and process compatibility on the mechanical behavior of composites. Subsequent studies should further clarify these mechanisms through surface modification and optimized dispersion processes.

As shown in Figure 7b, the total deformation of specimens doped with 0.25 wt.% CNF19 and CNF24 exhibits minimal variation compared to the blank group (difference < 5%). However, when the doping level increases to 0.50 wt.%, the total deformation at fracture of the cementitious composites increases significantly (+35% for CNF24-0.5). Notably, these stress–strain curves display a pattern of gradual ascent followed by rapid decline as the uniaxial tensile load continues to increase, contrasting sharply with the ductile response observed in short-cut carbon fiber-reinforced systems reported in reference [56]. This indicates that the incorporation of carbon nanofibers (CNFs) does not alter the brittle failure characteristics of cementitious composites under uniaxial tensile loading. This critical conclusion is directly validated by experimental observations: all specimens undergo rapid fracture upon reaching peak load, regardless of nanofiber addition. Although nanofiller doping cannot fundamentally modify the brittle nature of CRCCs, the ultimate deformation of composites doped with CNF19 and CNF24 remains 24.5% higher on average than that of undoped blank specimens. In other words, the uniaxial tensile load borne by a CRCC is jointly supported by both the cement matrix and nano-scale fibrous materials. As the load increases, microcracks start to form within the composite, at which point the one-dimensional nanofillers form bridging effects at the micro-scale [57], providing localized crack resistance and tensile reinforcement.

Figure 7c presents the stress–strain curves of CRCC specimens reinforced with different types of CFs under uniaxial tensile loading. The study reveals that the incorporation of CFs significantly enhances the ultimate strain of CRCCs (increased by 66.8%~140.8%), with the nonlinear characteristics of the curves becoming more pronounced as the fiber content increases. Notably, the tangent slope of the curves gradually decreases in the peak region, indicating that CFs effectively improve the toughness of CRCCs by delaying crack propagation. This phenomenon aligns with the conclusions from Zahra S [58] and Chen [59] regarding the toughness enhancement mechanisms in carbon fiber-reinforced concrete. However, the chopped carbon fiber-reinforced composites in this test exhibited limited post-peak load ductility, which may be attributed to the synergistic effects of fiber type, orientation, and interfacial bonding properties on the mechanical response of the composite material.

### 3.3. Uniaxial Tensile Strength

**Figure 8:**
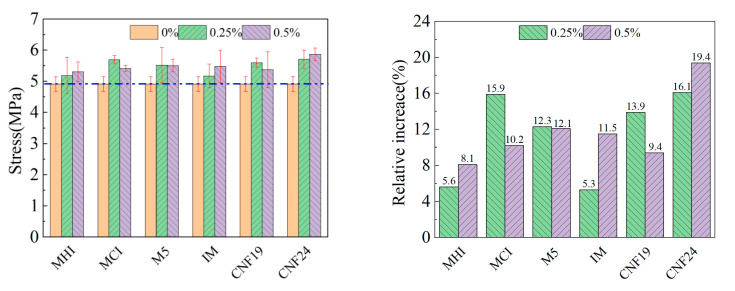
Uniaxial tensile strength of CRCCs with nano-scale fibrous materials.

The effect of nano-scale fibrous materials on the uniaxial tensile strength of CRCCs at 0.25 wt.% and 0.50 wt.% doping levels is shown in Figure 8. The dark blue horizontal line in the graph represents the stress baseline of PC group. CNF24 demonstrates the most significant enhancement of uniaxial tensile strength at 0.25 wt.% doping. Further increasing the doping amount to 0.50 wt.%, the uniaxial tensile strength of CRCCs incorporating MHI, MCI, M5, IM, and CNF19 are improved by 8.1%, 10.2%, 12.1%, 11.5%, and 9.4%, respectively, compared with the blank group. Also, CNF24 again exhibits the best enhancement effect of uniaxial tensile strength with an increase of 19.4%/0.95 MPa. However, it is also found that CRCCs doped with MCI and CNF19 show a decrease in uniaxial tensile strength as the filler content increases. In addition, the uniaxial tensile strength of CRCCs remains essentially unchanged when the doping of M5 rises from 0.25 wt.% to 0.50 wt.%. These phenomena may be attributed to the strong van der Waals forces causing some of the nanofillers to entangle and adhere to the surface of the cement particles at high dosages, forming an inhomogeneous slurry [55]. In this case, the pores and voids inside the CRCCs increase, and the matrix compactness decreases, thus weakening the reinforcing effect of the nanofillers [25]. Moreover, the differences in the optimal dispersion method, optimal sonication time, and optimal water-reducing agent dosage between the different nanofiller types may also lead to CRCCs exhibiting different uniaxial tensile strength development patterns.

**Figure 9:**
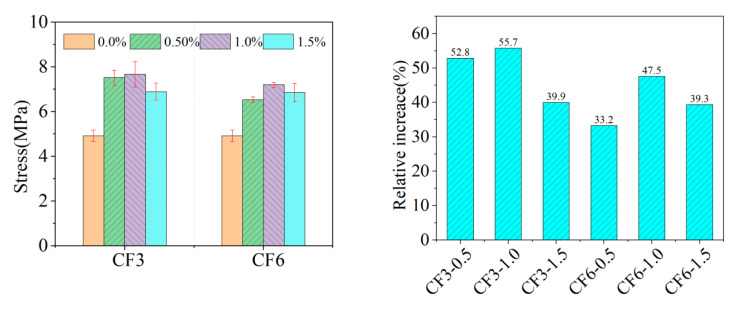
Uniaxial tensile strength of CRCCs with CFs.

In the investigation of CRCCs filled with CFs, the effects of CF3 and CF6 at the three doping levels of 0.50 vol.%, 1.0 vol.%, and 1.5 vol.% were examined. The research results are plotted in Figure 9. The addition of CF3 and CF6 at 0.50 vol.% doping can increase the uniaxial tensile strength of a CRCC by 52.8% and 33.2%, respectively, compared with that of blank group. Among them, the 3 mm-long CF3 demonstrates a relatively significant enhancement effect at this doping level, with an increase in uniaxial tensile strength by 1.95 MPa. The uniaxial tensile strength of CRCCs is further enhanced by CF3 and CF6, with an increase in doping up to 1.0 vol.% where the percentage increase can be up to 55.7% and 47.5%, respectively. At this doping level, the maximum increase in uniaxial tensile strength of 2.06 MPa is achieved by 3 mm-long CF3. However, the reinforcing effect of the two CFs shows a tendency to increase and then decrease as the dosage increases. When the doping content is increased to 1.5 vol.%, the uniaxial tensile strength of composites incorporating with CF3 and CF6 increases by 39.9% and 39.3%, respectively, compared with that of the blank group. This phenomenon may be related to the dispersion of fibers in the matrix and interfacial adhesion problems at high doping levels [39]. In addition, the length of the CFs also significantly affects the uniaxial tensile strength of CRCCs. Comparing 3 mm-long CF3 and 6 mm-long CF6, the former exhibits a higher uniaxial tensile strength reinforcement effect, especially at 0.50 vol.% and 1.0 vol.% dosage. These findings are of great significance for optimizing the formulation design of CRCCs. By accurately controlling the type, length, and doping amount of CFs, the mechanical properties of CRCCs can be effectively improved, which has practical guidance for maximizing the performance of CRCCs in engineering applications.

### 3.4. Fracture Strain Energy

**Figure 10:**
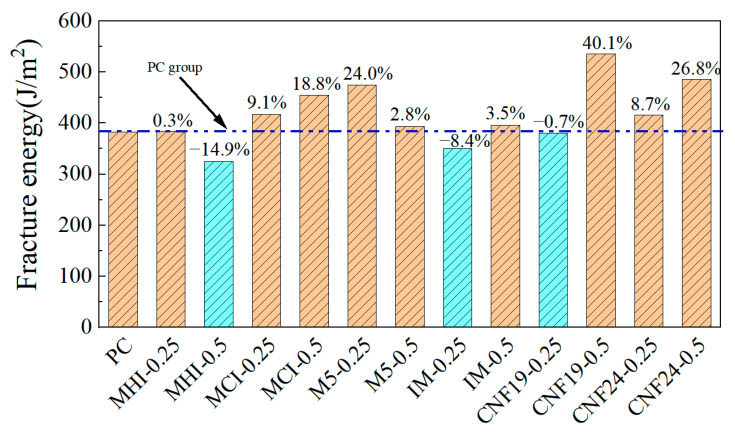
Fracture energy of CRCCs filled with nano-scale fibrous materials.

The fracture strain energies of CRCCs with different carbon nanofillers under a uniaxial tensile load were obtained by the corresponding data processing and calculation of the uniaxial tensile stress–strain curves, as followed in Figure 10. Under uniaxial tensile loading, except for the addition of 0.50 wt.% MHI, 0.25 wt.% IM, and 0.25 wt.% CNF19, the incorporation of other types and dosages of nanofillers can improve the fracture strain energy of the composites. The contribution of the four nanofillers to the fracture strain energy of CRCCs at high doping are greater than those at low doping, except for M5 and MHI. The significant deviation in the fracture strain energy of composites under the same test conditions is mainly due to differences in the appearance and development of microcracks within the CRCCs during the loading process, as well as apparent differences in the bridging effect and microcrack inhibition capabilities of the different types and dosages of nanofillers added [18]. It can also be found that the 0.50 wt.% dosage of CNF19 and CNF24 exhibits the most pronounced enhancement in the fracture strain energy of CRCCs.

**Figure 11:**
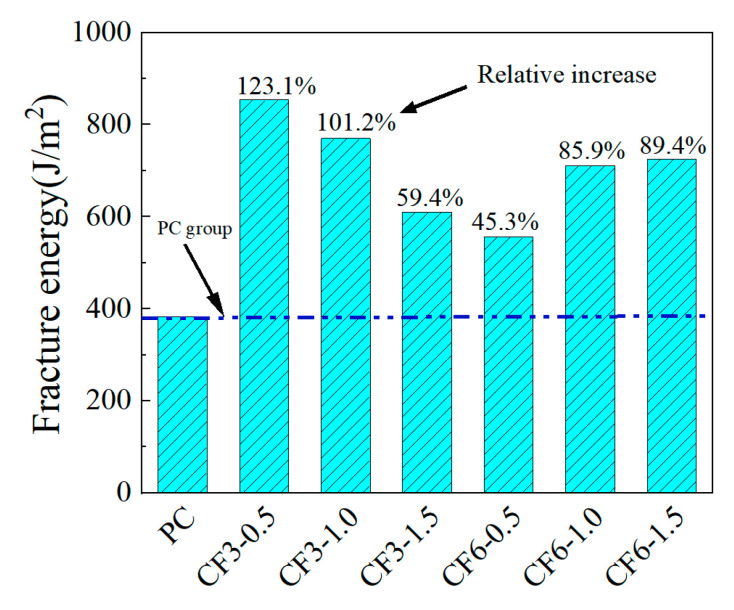
Fracture energy of CRCCs filled with CFs.

The fracture strain energies of different CRCCs doped with CFs under uniaxial tensile loading are presented in Figure 11. It is evident from Figure 11 that the fracture strain energy of CRCCs shows a general trend of gradual decrease with the increase in CF3 doping. The fracture strain energies of CRCCs doped with CF3 at 0.50 vol.%, 1.0 vol.%, and 1.5 vol.% dosage are separately 123.1%, 101.2%, and 59.4% higher than that of blank composites. Meanwhile, the improvements of the fracture strain energy of CRCCs doped with CF6 are 45.3%, 85.9%, and 89.4%, respectively, generally showing a trend of gradual increasing. Comprehensively, both CF3 and CF6 show significant improvements in fracture strain energy at 1.0 vol.% doping, which is consistent with the impact of CFs on the uniaxial tensile strength of CRCCs.

### 3.5. Conductivity

**Figure 12:**
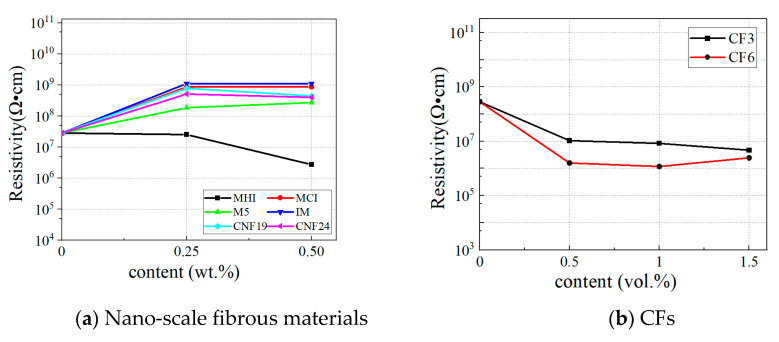
Volume resistivity of CRCCs.

Figure 12 shows the variation in the test volume resistivity of CRCCs with different carbon fillers. It can be observed from Figure 12 that the blank composite without carbon fibers exhibits a high resistivity of 3.56 × 10^8^ Ω·cm, indicating that the base composite itself has a high resistivity. In addition, the incorporation of nano-scale fibrous materials, except for MHI, fails to improve the electrical conductivity of CRCCs. On the contrary, incorporating these fillers further increases CRCC’s resistivity by an order of magnitude higher than the blank group. This may be due to the non-uniform distribution of these nanofillers in the CRCC matrix or their poor bonding with the matrix, resulting in the isolation of the conductive pathways. Nonetheless, it is gratifying to note that the incorporation of MHI demonstrates potential for improving the electrical conductivity of CRCCs. As the MHI doping increases from 0.25 wt.% to 0.50 wt.%, the resistivity of CRCCs decreases significantly, thus confirming that MHI effectively enhances the conductivity of composites. In other words, MHI is more prone to exhibiting conductivity and forming an effective conductive network than other nano-scale fibrous materials, thereby enhancing the electrical conductivity of CRCCs. Furthermore, as the doping amount of MHI increases the resistivity of CRCCs decreases, indicating that the conductive network has gone through the process of absence, emergence, and abundance.

According to Figure 12b, the volume resistivity of CRCCs is significantly decreased after doping with CFs. The resistivity of CRCCs doped with CF3 decreases continuously with increased doping. When the doping content reaches 1.5 vol.%, its resistivity can be reduced by 83.4%, representing a one-order-of-magnitude decrease compared with the blank group. While the resistivity of CRCCs doped with CF6 exhibits a trend of decreasing and then increasing with the increase in doping amount, when the doping amount is 1.0 vol.% their resistivity can be reduced by 95.8%, which is a two-order-of-magnitude decrease compared with the blank group. In addition, when the doping amount of the CFs is constant, the resistivity of the CRCCs doped with CF6 is smaller than that of the CRCCs doped with CF3, indicating that the larger the fiber length, the more pronounced the resistivity change. Moreover, it can be seen that with the doping of CFs, the resistivity of CRCCs decreases rapidly at first and then slowly. That is, there exists a critical value for fiber doping. Both CF3 and CF6 have a substantial decrease in resistivity at a fiber content of 0.5 vol.%, with a smaller change in resistivity after the content exceeds 0.5 vol.%. According to the theory of percolation threshold [55], the upper percolation threshold range of CF3 can be determined as 0~0.5 vol.% and the final percolation threshold is about 1.0~1.5 vol.%, whilst the upper percolation threshold of CF6 is 0~0.5 vol.% and the final percolation threshold is 0.5~1.0 vol.%.

The electrical conductivity of cementitious composites without carbon fibers primarily relies on ions within the cement hydration products, resulting in poor electrical conductivity of the material. The incorporation of CFs then can alter the conductivity mechanism of CRCCs. When a small number of CFs are doped, i.e., the fiber doping is below the percolation threshold, the CRCCs matrix primarily serves the function of conductivity. At this point, the small quantity of fibers are unable to form a connected conductive network, leading to an insignificant reduction in resistivity. With the increase in the doped number of CFs to the percolation threshold, the conductive fibers begin to play a major conductive role where they can achieve transitions within the matrix and generate a tunneling effect [38,60]. The conductive network is fully formed when the CFs in the matrix disperse uniformly, and then the resistivity of CRCC reaches a shallow level. However, when the amount of fiber doping is more than the infiltration threshold, the dispersion of carbon fibers becomes more difficult during mixing, resulting in an increased agglomeration effect. This phenomenon is more pronounced for fibers with greater lengths. Subsequently, further incorporation of carbon fibers has minimal impact on the electrical conductivity of CRCCs and may even cause it to decrease, as evidenced by the composites with 1.5 vol.% CF6 added.

### 3.6. The Relationship Between Fracture Strain Energy and Electrical Conductivity

CRCCs exhibit remarkable synergistic electromechanical properties, demonstrating a strong correlation between fracture strain energy and electrical conductivity. Comparative studies reveal distinct enhancement characteristics of carbon fibers at different scales; MHI can significantly reduce resistivity by two-orders-of-magnitude (from 3.56 × 10⁸ to 2.56 × 10^6^ Ω·cm) and yet provides limited improvement in fracture energy (approximately 15–20%). In contrast, CF6 at an optimal dosage of 1.0 vol.% simultaneously achieves an 85.9% increase in fracture strain energy and a 95.8% reduction in resistivity. This electromechanical synergy originates from the formation of a three-dimensional interconnected network, which enhances crack-bridging efficiency mechanically while establishing continuous conductive pathways electrically. Notably, excessive doping (>1.5 vol.%) leads to fiber agglomeration, causing a 15–20% resistivity rebound and 8.7% fracture strain energy degradation, primarily attributed to conductive pathway distortion and stress concentration intensification.

These findings provide critical guidelines for engineering applications. For structural components requiring both load-bearing capacity and self-monitoring functionality, the 1.0 vol.% CF6 doping formulation is recommended as it optimally balances an 85.9% fracture energy enhancement with a two-order-of-magnitude resistivity reduction. This performance optimization occurs near the percolation threshold (0.8–1.2 vol.%), where the fiber network achieves optimal connectivity for both mechanical stress transfer and electrical conduction. The research underscores the importance of precise dosage control and interfacial engineering in developing multifunctional cementitious composites for smart infrastructure applications.

## 4. Conclusions

In this study, CRCCs were fabricated using carbon fibers with different scales, and uniaxial tensile tests were conducted to investigate the effects of carbon fiber scale and dosage on the uniaxial tensile and electrical properties of cementitious composites. The main findings are as follows.

(1) All of the MWCNTs, CNFs, and CFs can significantly enhance the uniaxial tensile strength of cementitious composites at a suitable dosage. The enhancement effect is closely related to the geometric dimensions of the nanofillers, gradually decreasing in the order of carbon nanotubes, carbon nanofibers, and short-cut carbon fibers.
(2) There is a scaling effect in determining the fracture energy of cementitious composites in terms of strain by direct tensile test, and the larger the length of carbon fibers, the higher the fracture energy. The doping of macrofibers can significantly improve the uniaxial tensile fracture strain energy of cementitious composites, with a 0.5 vol.% incorporation of 3 mm-long short-cut carbon fibers having the most significant effect.
(3) Compared with other carbon nanofibers, the elongated hydroxyl-functionalized multi-walled carbon nanotubes are more likely to form an effective conductive network, thereby contributing to the enhancement of the electrical conductivity of a CRCC. The macro-short-cut carbon fibers have the most significant effect on enhancing the electrical conductivity of cementitious composites, and the longer the fibers, the more pronounced the improvement in resistivity. Additionally, the resistivity of cementitious composites displays a percolation phenomenon when short-cut carbon fibers are added within the range of 0.5 vol.% to 1.0 vol.%.

In future work, a hierarchical carbon nanotube-short cut carbon fiber reinforced system can be developed based on the nano–micron synergistic toughening mechanism to improve the fracture toughness and electrical conductivity of cementitious composites through interface optimization. Meanwhile, systematic evaluation and research on the self-sensing stability of composites should be conducted to achieve real-time monitoring of the structural health of intelligent infrastructure.

## Figures and Tables

**Table 1:** Chemical components of C [43].

Chemical Composition	SiO_2_	Al_2_O_3_	CaO	Fe_2_O_3_	MgO	SO_3_	Na_2_O
Quality score (%)	21.45	5.24	61.13	2.89	2.08	2.05	0.77

**Table 2:** Mineral components of C [43].

Chemical Composition	C_3_S	C_2_S	C_3_A	C_4_AF
Quality score (%)	46.62	26.32	8.99	8.78

**Table 3:** Chemical components of FA.

Chemical Composition	SiO_2_	Al_2_O_3_	CaO	Fe_2_O_3_	MgO	K_2_O	SO_3_	Na_2_O
Quality score (%)	49.05	22.48	9.36	5.95	5.41	1.48	0.54	0.98

**Table 4:** Properties of carbon fibers.

Type	Length (μm)	Outside Diameter (nm)	Inner Diameter (nm)	-COOH content (wt.%)	-OH Content (wt.%)	Specific Surface Area (m^2^/g)	Density (g/cm^3^)
MHI	10–30	<8	2–5	–	5.58	>400	2.1
MCI	10–30	<8	2–5	3.86	–	>400	2.1
M5	10–30	20–30	5–10	–	–	>110	2.1
IM	10–20	>50	–	–	–	>60	1.9
CNF19	50–200	150	–	–	–	15~24	1.9
CNF24	50–200	100	–	–	–	41	1.9
CF3	3000	7000	–	–	–	–	1.8
CF6	6000	7000	–	–	–	–	1.8

**Table 5:** Mix proportions of CRCC filled with nanofillers.

Type	Filler Dosage (wt.%)	C	FA	SF	QS	W	NSF
PC	–	0.8	0.2	0.25	1.1	0.3	0.011
CRCC	0.25%	0.798	0.2	0.25	1.1	0.3	0.011
	0.5%	0.796	0.2	0.25	1.1	0.3	0.011

**Table 6:** The peak strain and peak stress of CRCCs under uniaxial tension load.

Types		Length (μm)	Strain (με)				Stress (MPa)			
			0.25 wt.%		0.5 wt.%		0.25 wt.%		0.5 wt.%	
Nanoscale	MHI	10–30	121.36		115.84		5.58		5.73	
	MCI	10–30	125.93		145.74		5.53		5.40	
	M5	10–30	164.72		146.00		6.03		5.64	
	IM	10–20	111.28		112.43		5.67		6.09	
	CNF19	50–200	124.86		149.80		5.74		6.25	
	CNF24	50–200	134.56		176.38		6.03		5.86	
Types		Length (μm)	0.5 vol.%	1.0 vol.%		1.5 vol.%	0.5 vol.%	1.0 vol.%		1.5 vol.%
Macro scale	CF3	3000	315.23	212.83		202.99	6.98	7.88		7.38
	CF6	6000	221.49	255.86		218.30	6.72	7.11		7.30

## Data Availability

The original contributions presented in this study are included in the article. Further inquiries can be directed to the corresponding authors.

## References

[1] Shi C.J., He W., Wu Z., Wu L., Zhu D., Huang Z., Zhang J. (2015). Research progress on the effect of fiber on the mechanical properties of UHPC. Bull. Chin. Ceram. Soc..

[2] Gong J.H., Ma Y., Fu J., Hu J., Ouyang X., Zhang Z., Wang H. (2022). Utilization of fibers in ultra-high performance concrete: A review. Compos. Part B-Eng..

[3] Deng Y.L., Zhang Z.H., Shi C.J., Wu Z.M., Zhang C.H. (2023). Steel fiber-matrix interfacial bond in ultra-high performance concrete: A review. Engineering.

[4] Hager I., Mróz K. (2019). Role of polypropylene fibres in concrete spalling risk mitigation in fire and test methods of fibres effectiveness evaluation. Materials.

[5] He J.Y., Chen W.Z., Zhang B.S., Yu J.J., Liu H. (2021). The mechanical properties and damage evolution of UHPC reinforced with glass fibers and high-performance polypropylene fibers. Materials.

[6] Patchen A., Young S., Penumadu D. (2023). An investigation of mechanical properties of recycled carbon fiber reinforced ultra-high-performance concrete. Materials.

[7] Zollo R.F. (1997). Fiber-reinforced concrete: An overview after 30 years of development. Cem. Concr. Compos..

[8] Ren L., Fang Z., Wang K. (2019). Design and behavior of super-long span cable-stayed bridge with CFRP cables and UHPC members. Compos. Part B-Eng..

[9] Gesoglu M., Güneyisi E., Muhyaddin G.F., Asaad D.S. (2016). Strain hardening ultra-high performance fiber reinforced cementitious composites: Effect of fiber type and concentration. Compos. Part B-Eng..

[10] Li P.P., Sluijsmans M.J.C., Brouwers H.J.H., Yu Q.L. (2020). Functionally graded ultra-high performance cementitious composite with enhanced impact properties. Compos. Part B-Eng..

[11] Feng J., Gao X., Li J., Dong H., Yao W., Wang X., Sun W. (2019). Influence of fiber mixture on impact response of ultra-high-performance hybrid fiber reinforced cementitious composite. Compos. Part B-Eng..

[12] Su Y., Li J., Wu C.Q., Wu P.T., Li Z.X. (2016). Influences of nano-particles on dynamic strength of ultra-high performance concrete. Compos. Part B-Eng..

[13] Chung D.D.L. (2004). Use polymers for cement-based structural materials. J. Mater. Sci..

[14] Liu B., Zhou J.K., Wen X.Y., Hu X., Deng Z.H. (2020). Mechanical properties and constitutive model of carbon fiber reinforced coral concrete under uniaxial compression. Constr. Build. Mater..

[15] Shi T., Li Z.X., Guo J., Gong H., Gu C.P. (2019). Research progress on CNTs/CNFs-modified cement-based composites—A review. Constr. Build. Mater..

[16] Wang D.N., Wang X.Y., Qiu L.S., Ye H.L., Maimaitituersun N., Han B.G. (2023). Effect of nickel-coated carbon nanotubes on the tensile behaviors of ultra-high performance concrete (UHPC): Insights from experiments and molecular dynamic simulations. J. Mater. Sci..

[17] Tyson B.M., Abu Al-Rub R.K., Yazdanbakhsh A., Grasley Z. (2011). Carbon nanotubes and carbon nanofibers for enhancing the mechanical properties of nanocomposite cementitious materials. J. Mater. Civ. Eng..

[18] Abu Al-Rub R.K., Ashour A.I., Tyson B.M. (2012). On the aspect ratio effect of multi-walled carbon nanotube reinforcements on the mechanical properties of cementitious nanocomposites. Constr. Build. Mater..

[19] Li S., Zhang Y., Cheng C., Wei H., Du S., Yan J. (2021). Surface-treated carbon nanotubes in cement composites: Dispersion, mechanical properties and microstructure. Constr. Build. Mater..

[20] Ahmed H., Bogas J.A., Guedes M., Pereira M.F.C. (2019). Dispersion and reinforcement efficiency of carbon nanotubes in cementitious composites. Mag. Concr. Res..

[21] Onuaguluchi O., Panesar D.K., Sain M. (2014). Properties of nanofibre reinforced cement composites. Constr. Build. Mater..

[22] Konsta-Gdoutos M.S., Danoglidis P.A., Falara M.G., Nitodas S.F. (2017). Fresh and mechanical properties, and strain sensing of nanomodified cement mortars: The effects of MWCNT aspect ratio, density and functionalization. Cem. Concr. Compos..

[23] Du Y., Yang J., Thomas B.S., Li L., Li H., Nazar S. (2020). Hybrid graphene oxide/carbon nanotubes reinforced cement paste: An investigation on hybrid ratio. Constr. Build. Mater..

[24] Zhou C., Li F., Hu J., Ren M., Wei J., Yu Q. (2017). Enhanced mechanical properties of cement paste by hybrid graphene oxide/carbon nanotubes. Constr. Build. Mater..

[25] Xu L., Liu K., Wang R. (2024). Effect and mechanism of carbon nanotubes on the properties of polyacrylate/cement composite cementitious materials. Constr. Build. Mater..

[26] Arrechea S., Guerrero-Gutiérrez E.M.A., Velásquez L., Cardona J., Posadas R., Callejas K., Torres S., Díaz R., Barrientos C., García E. (2020). Effect of additions of multiwall carbon nanotubes (MWCNT, MWCNT-COOH, and MWCNT-Thiazol) in the mechanical compression properties of a cement-based material. Materialia.

[27] Li G.Y., Wang P.M., Zhao X. (2005). Mechanical behavior and microstructure of cement composites incorporating surface-treated multi-walled carbon nanotubes. Carbon.

[28] Wang Z.K., Yu J., Li G.Y., Zhang M., Leung C.K.Y. (2019). Corrosion behavior of steel rebar embedded in hybrid CNTs-OH/polyvinyl alcohol modified concrete under accelerated chloride attack. Cem. Concr. Compos..

[29] Abu Al-Rub R.K., Tyson B.M., Yazdanbakhsh A., Grasley Z. (2012). Mechanical properties of nanocomposite cement incorporating surface-treated and untreated carbon nanotubes and carbon nanofibers. J. Nanomech. Micromech..

[30] Wang T.J., Xu J.Y., Meng B.X., Peng G. (2020). Experimental study on the effect of carbon nanofiber content on the durability of concrete. Constr. Build. Mater..

[31] Rizvi H.R., Khattak M.J., Madani M., Khattab A. (2016). Piezoresistive response of conductive Hot Mix Asphalt mixtures modified with carbon nanofibers. Constr. Build. Mater..

[32] Konsta-Gdoutos M.S., Danoglidis P.A., Shah S.P. (2019). High modulus concrete: Effects of low carbon nanotube and nanofiber additions. Theor. Appl. Fract. Mech..

[33] Konsta-Gdoutos M.S., Batis G., Danoglidis P.A., Zacharopoulou A.K., Zacharopoulou E.K., Falara M.G., Shah S.P. (2017). Effect of CNT and CNF loading and count on the corrosion resistance, conductivity, and mechanical properties of nano modified OPC mortars. Constr. Build. Mater..

[34] Li L., Wang B., Hubler M.H. (2022). Carbon nanofibers (CNFs) dispersed in ultra-high performance concrete (UHPC): Mechanical property, workability and permeability investigation. Cem. Concr. Compos..

[35] Gao X.J., Wang H., Li S.X., Lu L., Mo Y.L. (2015). Piezoresistive effect of carbon nanofiber concrete exposed to different environmental conditions. Rom. J. Mater..

[36] Wang T., Xu J., Bai E., Luo X., Chen H., Liu G., Chang S. (2019). Study on the effects of carbon fibers and carbon nanofibers on electrical conductivity of concrete. IOP Conf. Ser. Earth Environ. Sci..

[37] Hao Y.L., Shi C., Bi Z., Lai Z., She A., Yao W. (2023). Recent advances in properties and applications of carbon fiber-reinforced smart cement-based composites. Materials.

[38] Chung D.D.L. (1998). Self-monitoring structural materials. Mater. Sci. Eng. R Rep..

[39] Pakravan H.R., Latifi M., Jamshidi M. (2017). Hybrid short fiber reinforcement system in concrete: A review. Constr. Build. Mater..

[40] Mastali M., Dalvand A., Sattarifard A. (2017). The impact resistance and mechanical properties of the reinforced self-compacting concrete incorporating recycled CFRP fiber with different lengths and dosages. Compos. Part B-Eng..

[41] Abellán-García J. (2022). Tensile behavior of recycled-glass-UHPC under direct tensile loading. Case Stud. Constr. Mater..

[42] Esmaeili J., Romouzi V., Kasaei J., Andalibi K. (2023). An investigation of durability and the mechanical properties of ultra-high performance concrete (UHPC) modified with economical graphene oxide nano-sheets. J. Build. Eng..

[43] Sun T., Wang X., Maimaitituersun N., Dong S., Li L., Han B. (2024). Synergistic effects of steel fibers and steel wires on uniaxial tensile mechanical and self-sensing properties of UHPC. Constr. Build. Mater..

[44] Hu T., Jing H.W., Li L., Yin Q., Sh X.S., Zhao Z.L. (2019). Humic acid assisted stabilization of dispersed single-walled carbon nanotubes in cementitious composites. Nano Rev..

[45] Meng W., Khayat K.H. (2016). Mechanical properties of ultra-high-performance concrete enhanced with graphite nanoplatelets and carbon nanofibers. Compos. Part B-Eng..

[46] Liu C., He X., Deng X., Wu Y., Zheng Z., Liu J., Hui D. (2020). Application of nanomaterials in ultra-high performance concrete: A review. Nano Rev..

[47] Han B.G., Zhang K., Yu X., Kwon E., Ou J.P. (2012). Fabrication of piezoresistive cnt/cnf cementitious composites with superplasticizer as dispersant. J. Mater. Civ. Eng..

[48] (2019). Standard for Test Methods of Concrete Physical and Mechanical Properties.

[49] (2009). Standard Test Methods for Fiber Reinforced Concrete.

[50] Khalilpour S., BaniAsad E., Dehestani M. (2019). A review on concrete fracture energy and effective parameters. Cem. Concr. Res..

[51] Han X.Y., Wang H., Gao H., Zheng J., Yu R.C., Wang Z., Wu Z. (2024). Fracture energy of concrete after sustained loading. Eng. Fract. Mech..

[52] Jia M.D., Wu Z.M., Yu R.C., Zhang X.X. (2021). Residual fracture energy of concrete suffering from fatigue loading. Eng. Fract. Mech..

[53] (2007). Test for Resistivity of Metallic Materials.

[54] Chen Z., Lim J.L.G., Yang E. (2016). Ultra high performance cement-based composites incorporating low dosage of plasma synthesized carbon nanotubes. Mater. Des..

[55] Ruschau G.R., Yoshikawa S., Newnham R.E. (1992). Resistivities of conductive composites. J. Appl. Phys..

[56] Lu L., Suliman M., Xia W. (2024). Reversed Cyclic Behavior of Carbon Nanofiber-Reinforced Concrete Shear Walls. Materials.

[57] Li Z., He Z.J., Zhou Y.C., Tang Y., Chen Y.F., Jin T. (2020). Effect of Dimethyl Sulfoxide in Hydrophobic Modification of Cotton Filter Cloth by ARGET-ATRP Mechanism. Mater. Sci. For..

[58] Tabatabaei Z.S., Volz J.S. (2014). Comparative impact behavior of four long carbon fiber reinforced concretes. Mater. Des..

[59] Chen Z.Y., Yang J. (2021). Experimental Study on Dynamic Splitting Characteristics of Carbon Fiber Reinforced Concrete. Materials.

[60] Wang D.Y., Dong S., Wang X., Maimaitituersun N., Shao S., Yang W., Han B. (2022). Sensing performances of hybrid steel wires and fibers reinforced ultra-high performance concrete for in-situ monitoring of infrastructures. J. Build. Eng..

